# SHMT2 is essential for mammalian preimplantation embryonic development through *de novo* biosynthesis of nucleotide metabolites

**DOI:** 10.1016/j.omtn.2025.102499

**Published:** 2025-03-05

**Authors:** Mingze Shi, Yingxue Huai, Tiantian Deng, Chuanxin Zhang, Jinzhu Song, Jiawei Wang, Yiwen Zhang, Zi-Jiang Chen, Han Zhao, Keliang Wu, Boyang Liu

**Affiliations:** 1 State Key Laboratory of Reproductive Medicine and Offspring Health, Center for Reproductive Medicine, Institute of Women, Children and Reproductive Health, Shandong University, Jinan, Shandong 250012, China; 2 National Research Center for Assisted Reproductive Technology and Reproductive Genetics, Shandong University, Jinan, Shandong 250012, China; 3 Key Laboratory of Reproductive Endocrinology (Shandong University), Ministry of Education, Jinan, Shandong 250012, China; 4 Shandong Key Laboratory of Reproductive Medicine, Jinan, Shandong 250012, China; 5 Research Unit of Gametogenesis and Health of ART-Offspring, Chinese Academy of Medical Sciences (No. 2021RU001), Jinan, Shandong 250012, China

**Keywords:** MT: Oligonucleotides: Therapies and Applications, SHMT2, nucleotide, metabolism, embryo, development

## Abstract

Assisted reproductive technology (ART) is used widely and efficiently to treat infertility. During the ART procedure, one of the main factors affecting the success rate is abnormal development of preimplantation embryos. The establishment and maintenance of developmental competence are precisely regulated at different levels, while minor errors at early stages may result in adverse outcomes, including developmental arrest and implantation failure. As one of the major inputs, the regulatory mechanisms of metabolites in embryonic development are less known. In this study, we investigated the functional relevance of the metabolic enzyme serine hydroxymethyltransferase 2 (SHMT2) and deoxyribonucleotide (dNTP) metabolites in mouse preimplantation embryonic development. By using a well-characterized SHMT2 inhibitor, SHMT-IN-2, we effectively inhibited the catalytic activity of the SHMT2 enzyme, which led to developmental arrest at the pronuclear stage of the embryo. A low-input liquid chromatography-tandem mass spectrometry (LC-MS/MS) method was developed and applied for detecting dNTP content in embryos. We found that SHMT2 inhibition led to an insufficient dTTP supply and replication stress during the first mitotic cleavage, thereby causing failure of pronuclear fusion and developmental arrest. Our findings demonstrate a specific mechanism where, apart from building blocks of DNA, the availability of dNTPs contributes to the control of mouse preimplantation embryonic development.

## Graphical abstract

**Figure:**
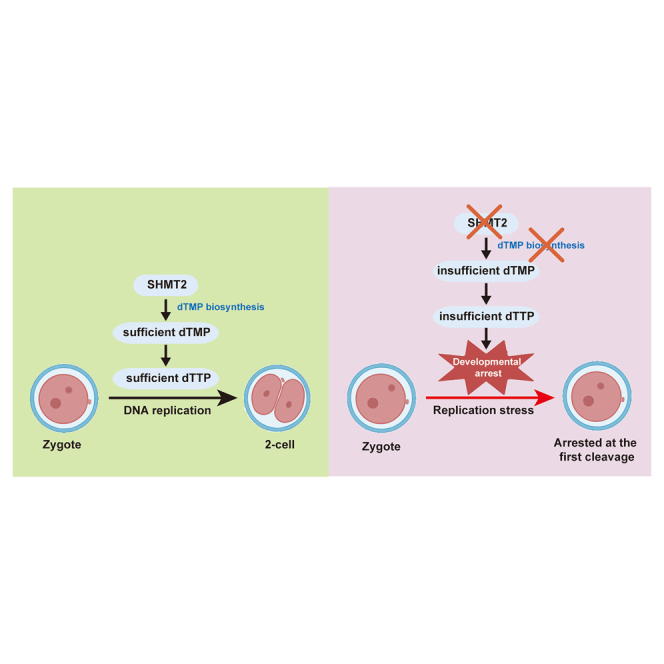

## Teaser

In this research article, Liu and colleagues demonstrate a specific mechanism where, apart from substrates of DNA biosynthesis, the availability of nucleotide metabolites contributes to the control of mammalian preimplantation embryonic development.

## Introduction

During mammalian preimplantation embryonic development, cell cleavage proceeds concurrently with fast DNA replication and mitosis. This fast replication demands high consumption of deoxyribonucleotides (dNTPs), the substrates of DNA synthesis. The biosynthesis and dynamics of dNTPs are essential for early embryonic development, when the genome is duplicated rapidly and dNTPs are consumed exponentially in each cell cleavage. Disruption of DNA synthesis prevents S phase completion and therefore hampers embryonic development. In the clinic, approximately 10% of human embryos produced by *in vitro* fertilization (IVF) or intracytoplasmic sperm injection (ICSI) are arrested in the early embryonic stage,^1^ but the pathological relevance remains elusive.

Parts of dNTPs are deposited in the egg during oogenesis, while parts are synthesized *de novo* by the embryo. The metabolic enzymes serine hydroxymethyltransferase 1/2 (SHMT1/2) play an important role in folate metabolism, which converts serine into glycine and tetrahydrofolate (THF) into 5,10-methylenetetrahydrofolate (CH_2_-THF), and CH_2_-THF is a substrate for the biosynthesis of dTMP from dUMP and, thus, the production of dTTP. This reaction is one of the essential steps of folate metabolism, which has been proven to be crucial for human fertility. Key metabolites in the folate cycle maintain the metabolism of nucleotides, proteins and lipids, and fuel methyltransferase reactions that shape the epigenetic landscape. Some folate metabolic enzymes have been proven to have functions in embryogenesis. For instance, PHGDH disruption results in mouse embryonic lethality,^2^ DHFR is required for zebrafish development,^3^ and MTHFR contributes to bovine blastocyst development.^4^ However, our understanding of folate metabolism in embryogenesis is limited so far.

Embryos lacking SHMT1/2 are predicted to be unable to *de novo* synthesize dTMP, but the cytosolic form SHMT1 is not essential for mice, whereas loss of the mitochondrial form SHMT2 is embryonic lethal, suggesting that the mitochondrial form may be more important in early development through compensation for the cytosolic form.^5^^,^^6^^,^^7^^,^^8^ Knockdown of maternal *Shmt2* in the oocyte results in developmental arrest; only 30% of embryos derived from knockdown oocytes develop to the 2-cell stage on day 1.5.^9^ Given that H3K4me3 levels are reduced in the maternal pronucleus (PN) upon SHMT2 depletion, SHMT2 activity is specifically required during meiotic resumption. However, the regulatory mechanism of SHMT2 in mammalian embryonic development is unclear.

In this study, we investigated the functional relevance of the SHMT2 enzyme and dNTP metabolites in mouse preimplantation embryonic development. The SHMT-IN-2 inhibitor was employed to inhibit the catalytic activity of the SHMT2 enzyme, which led to an insufficient dTTP supply and replication stress during the first embryonic cleavage cycle, resulting in developmental arrest prior to the 2-cell stage. We therefore demonstrate a specific mechanism where, apart from substrates of DNA biosynthesis, the availability of nucleotide metabolites contributes to the regulation of mammalian preimplantation development.

## Results

### Essential functions of SHMT2 in mammalian preimplantation embryonic development

**Figure 1:**
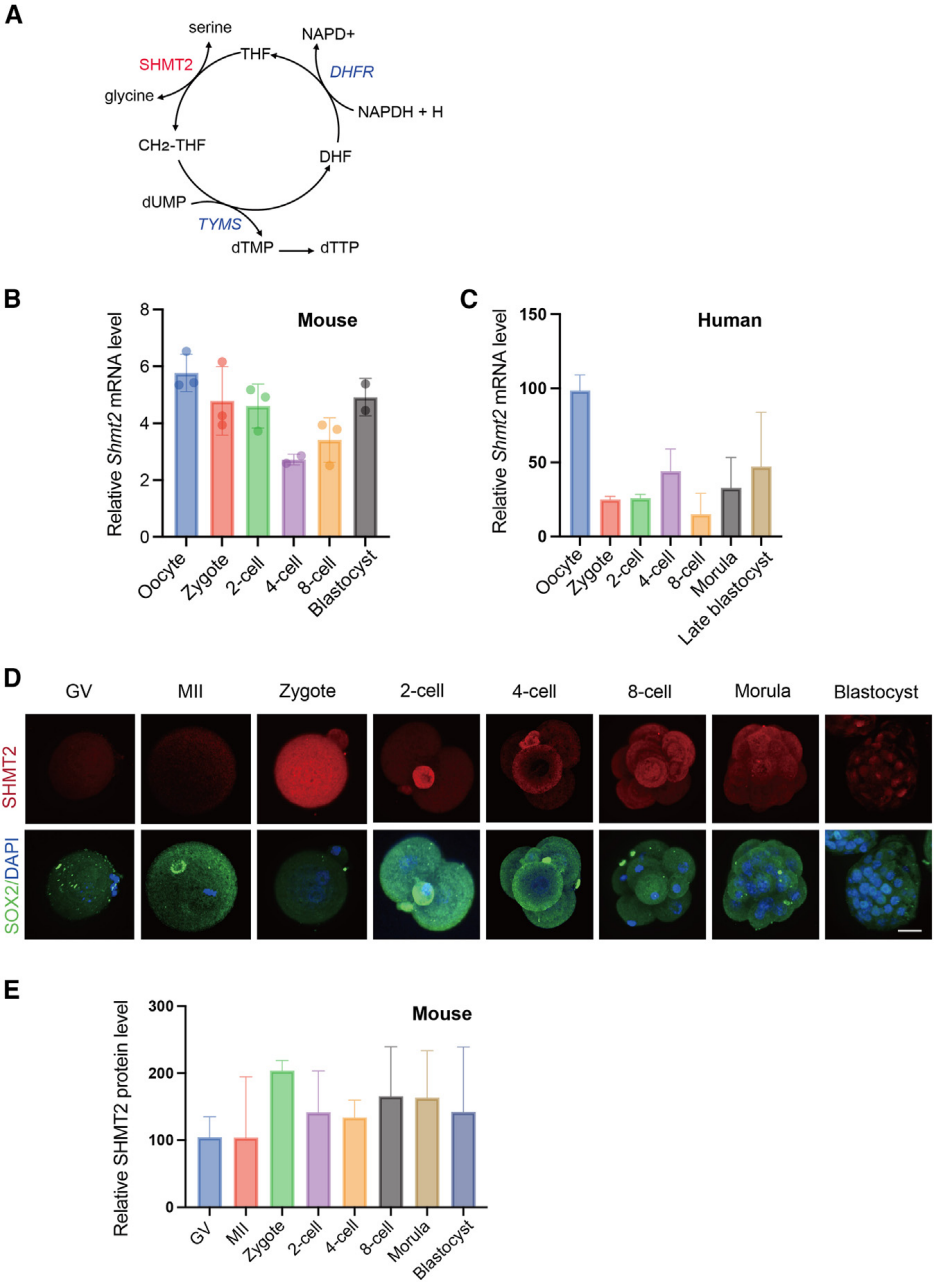
Functions of SMHT2 during early embryonic development (A) Overview of the *de novo* biosynthesis pathway of dTTP as part of the folate metabolic pathway. The key enzyme SHMT2 is highlighted in red. Other enzyme encoding genes are highlighted in blue. THF, tetrahydrofolate; CH_2_-THF, 5,10-methylenetetrahydrofolate; SHMT, serine hydroxymethyltransferase. (B and C) Analysis of previously published data^10^^,^^11^ from the RNA-seq assay of *Shmt2* mRNA in human and mouse oocytes and preimplantation embryos. The results of relative quantification are presented as the mean ± SEM (*n* = 3). (D) Immunostaining of SHMT2 protein and the persistent marker SOX2 at different stages during mouse preimplantation embryonic development. Scale bar, 25 μm. (E) Quantification of relative SHMT2 protein fluorescence intensity from immunostained mouse embryos at different developmental stages. The results of relative quantification are presented as the mean ± SEM (*n* = 3).

SHMT2 is a pyridoxal phosphate-dependent enzyme that is involved in the biosynthesis of dTTP and evolutionarily highly conserved among species (Figures 1A and S1A–S1C). To compare the dynamics of *Shmt2* mRNA levels in human and mouse preimplantation development, we analyzed previously published data from the RNA sequencing (RNA-seq) assays.^10^^,^^11^ Mouse oocytes stored the most *Shmt2* mRNA, indicating an excessive maternal mRNA pool. The mRNA level degraded after fertilization until 4-cell stage and increased from 8-cell to blastocyst stage, while the increase was later than zygotic genome activation (ZGA). In humans, SHMT2 also showed the highest mRNA levels in oocytes, and the levels in the zygote, 2-cell, and 8-cell stages were relatively low but higher in the 4-cell stage (Figures 1B and 1C). This may be related to the difference of ZGA timing between mouse and human.

We collected oocytes from the GV and MII stage and embryos from zygote to blastocyst by IVF to observe the dynamics of the SHMT2 protein. Semi-quantification of immunofluorescence staining showed that, in oocytes, the SHMT2 protein level was low (Figures 1D and 1E), while redundant mRNAs were stored (Figure 1B). Thereafter, SHMT2 protein levels were highest in the zygote stage, declined from the 2-cell to the 4-cell stage, and increased in the 8-cell stage (Figures 1D and 1E). The tendency was concomitant with *Shmt2* mRNA levels. This may explain the fact that homozygous *Shmt2*-deficient mice obtained from heterozygous parents showed lethality after 13.5 days post coitum.^5^^,^^6^ Homozygous *Shmt2*-deficient zygotes could still utilize maternal SHMT2 enzyme loaded by the heterozygous mother, despite not sufficient.

### SHMT2 inhibition leads to developmental arrest at the pronuclear stage

**Figure 2:**
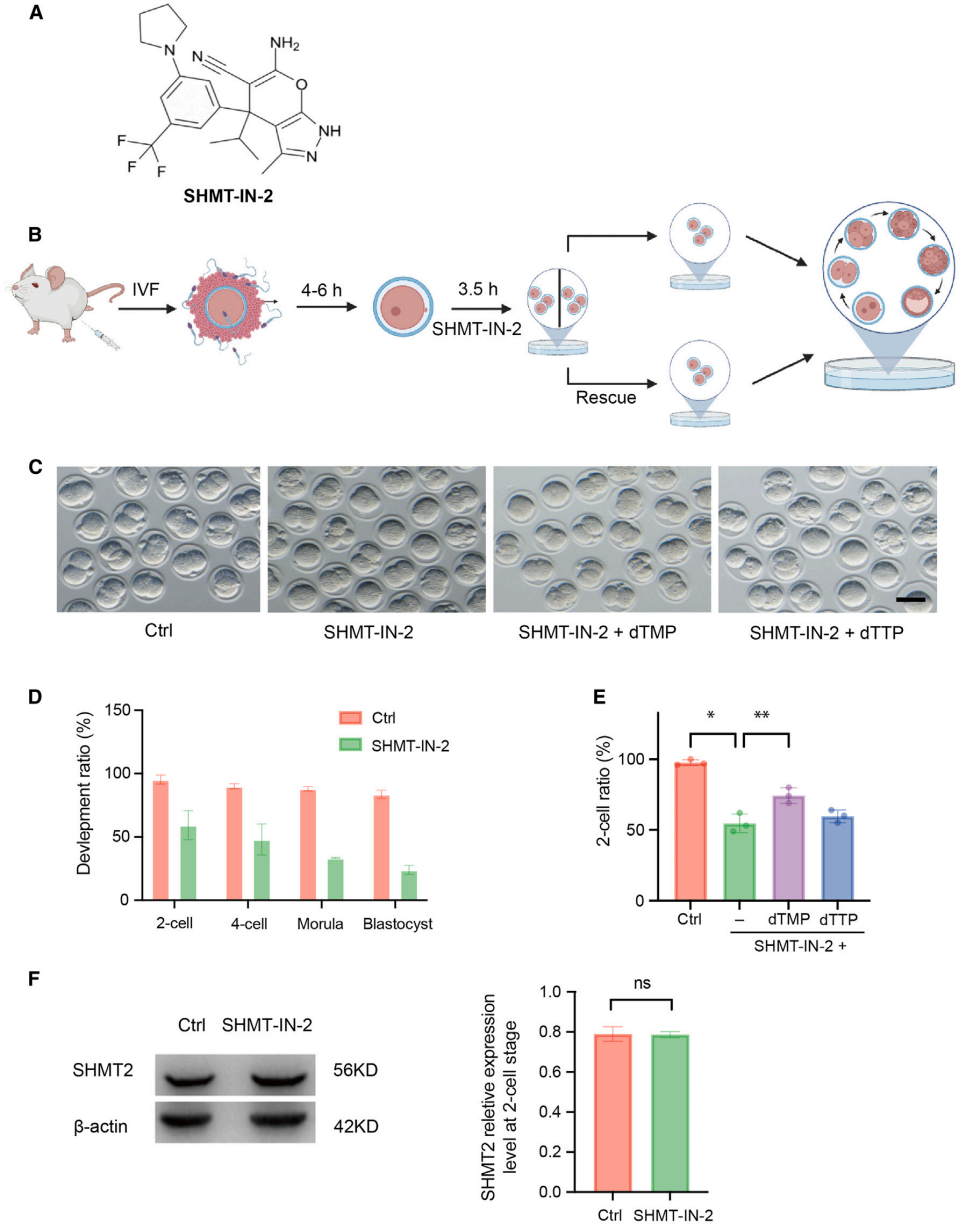
Inhibition of SHMT2 results in developmental arrest at the pronuclear stage (A) The chemical structure of the SHMT2 molecular inhibitor SHMT-IN-2. (B) Schematic overview of mouse embryo SHMT-IN-2 treatment and rescue experiments. (C) Representative images from bright-field microscopy of *in vitro*-cultured mouse embryos treated with SHMT-IN-2, SHMT-IN-2 and 50 μM dTMP, and SHMT-IN-2 and 50μM dTTP. Arrested embryos were observed after the SHMT-IN-2 treatment. Scale bar, 100 μm. (D) Developmental rates of control and SHMT-IN-2-treated embryos at different stages. Data are from three independent experiments and are presented as the mean ± SEM. (E) 2-cell rates of control, SHMT-IN-2-treated, and SHMT-IN-2 and dTMP/dTTP-supplemented embryos. Data are from three independent experiments and are presented as the mean ± SEM. ∗*p* < 0.05, ∗∗*p* < 0.01 (Student’s t test). (F) Western blot analysis and quantification showing that SHMT2 protein levels in 2-cell embryos did not change after inhibitor treatment. *n* = 3. 10 embryos per lane. kD, kilodalton; ns, not significant.

To examine the function of SHMT2 during embryogenesis, we inhibited SHMT2 maternally. Since both SHMT2 protein and mRNA are present at a high level in the zygote, rather than small interfering RNA injection, we turned to chemical inhibition of SHMT2 protein by a widely used and well-characterized inhibitor, SHMT-IN-2 (Figure 2A).^12^ After fertilization, zygotes were treated with the SHMT2 inhibitor for 3.5 h, and the development was assayed (Figure 2B). Approximately 50% of the embryos treated with the SHMT2 inhibitor arrested at the pronuclear stage, failing to reach the 2-cell stage. Additionally, only 25% of the treated embryos developed to the blastocyst stage, which was significantly lower than for the control group (Figures 2C–2E). This result is consistent with RNA interference experiments.^9^ SHMT-IN-2 did not affect SHMT2 protein levels in 2-cell embryos but, rather, changed the catalytic activity of the enzyme (Figures 2F and S2C). Unexpectedly, addition of dTTP did not rescue the developmental arrest caused by the SHMT2 inhibition, whereas the development was partially rescued by the supplementation of dTMP, the upstream intermediate metabolite of dTTP (Figures 2C–2E). This may indicate that dTMP plays other roles apart from being the substrate of dTTP synthesis.

**Figure 3:**
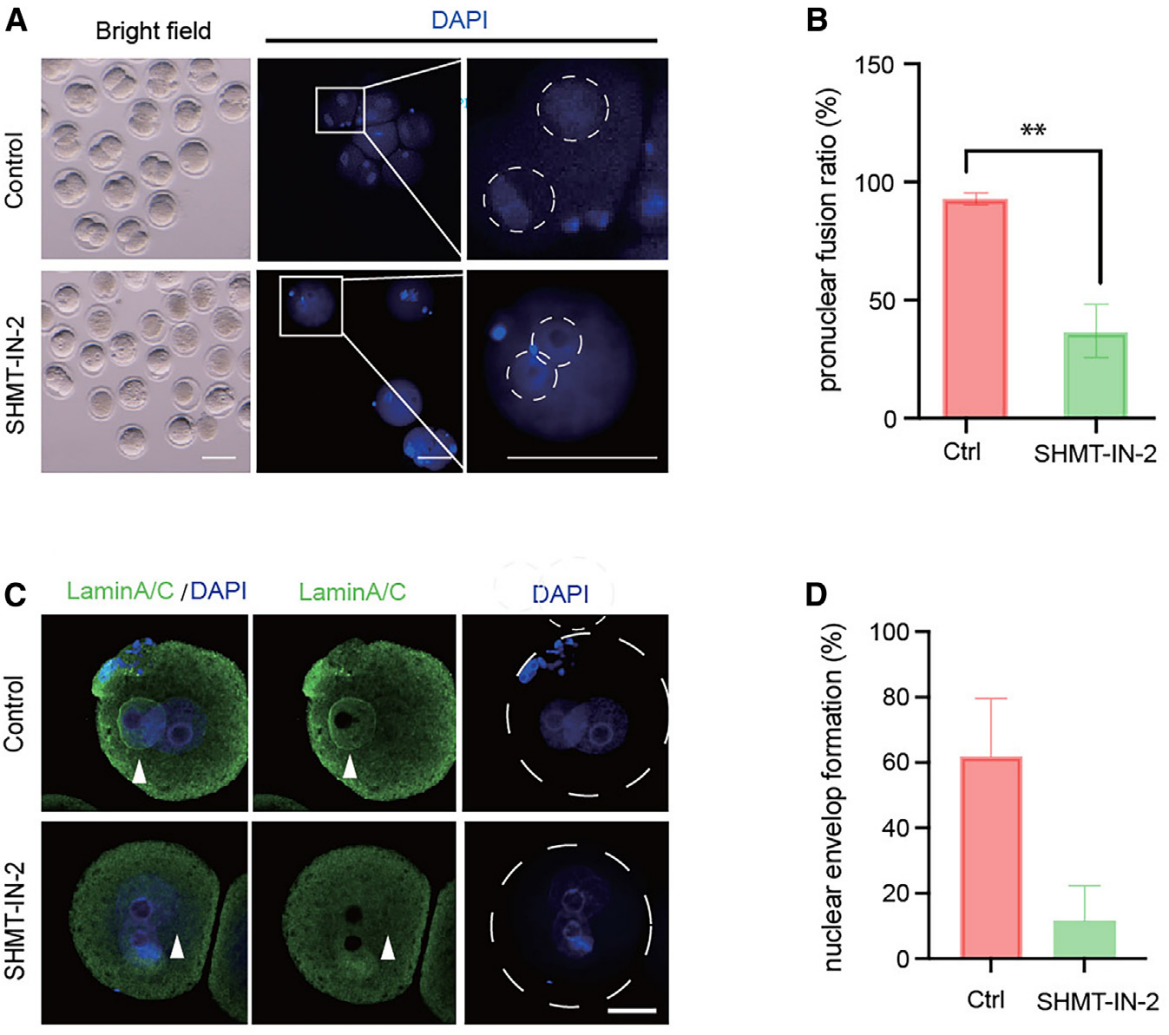
Nuclear envelope formation is impaired by SHMT2 inhibition (A) Representative bright-field and DAPI fluorescence images showing nuclear DNA in 2-cell-stage control and developmentally arrested embryos. Scale bar, 100 μm. Nuclei are magnified in white boxes and marked by white circles. (B) Quantification of pronuclear fusion rate in control and SHMT-IN-2-treated embryos. Data are from three independent experiments. ∗∗*p* < 0.01 (Student’s t test). (C) Representative immunofluorescence images showing LaminA/C and DAPI staining in late-zygote-stage control and developmentally arrested embryos. White arrowheads mark the position of the nuclear membrane. Scale bar, 20 μm. (D) Quantification of nuclear envelope formation rate in the control and SHMT-IN-2-treated embryos. Data are from three independent experiments and are presented as the mean ± SEM.

We stained nuclear DNA and the envelope to confirm the developmental arrest phenotype by SHMT2 inhibition. Pronuclear fusion failure was detected by DAPI staining in more than 50% of SHMT2-inhibited embryos (Figures 3A and 3B). Furthermore, the formation of the nuclear envelope was also impaired with the absence of maternal SHMT2, suggesting the occurrence of DNA replication stalling among two pronuclei (Figures 3C and 3D).

### SHMT2 inhibition-induced developmental arrest is caused by dTTP depletion and replication stress

**Figure 4:**
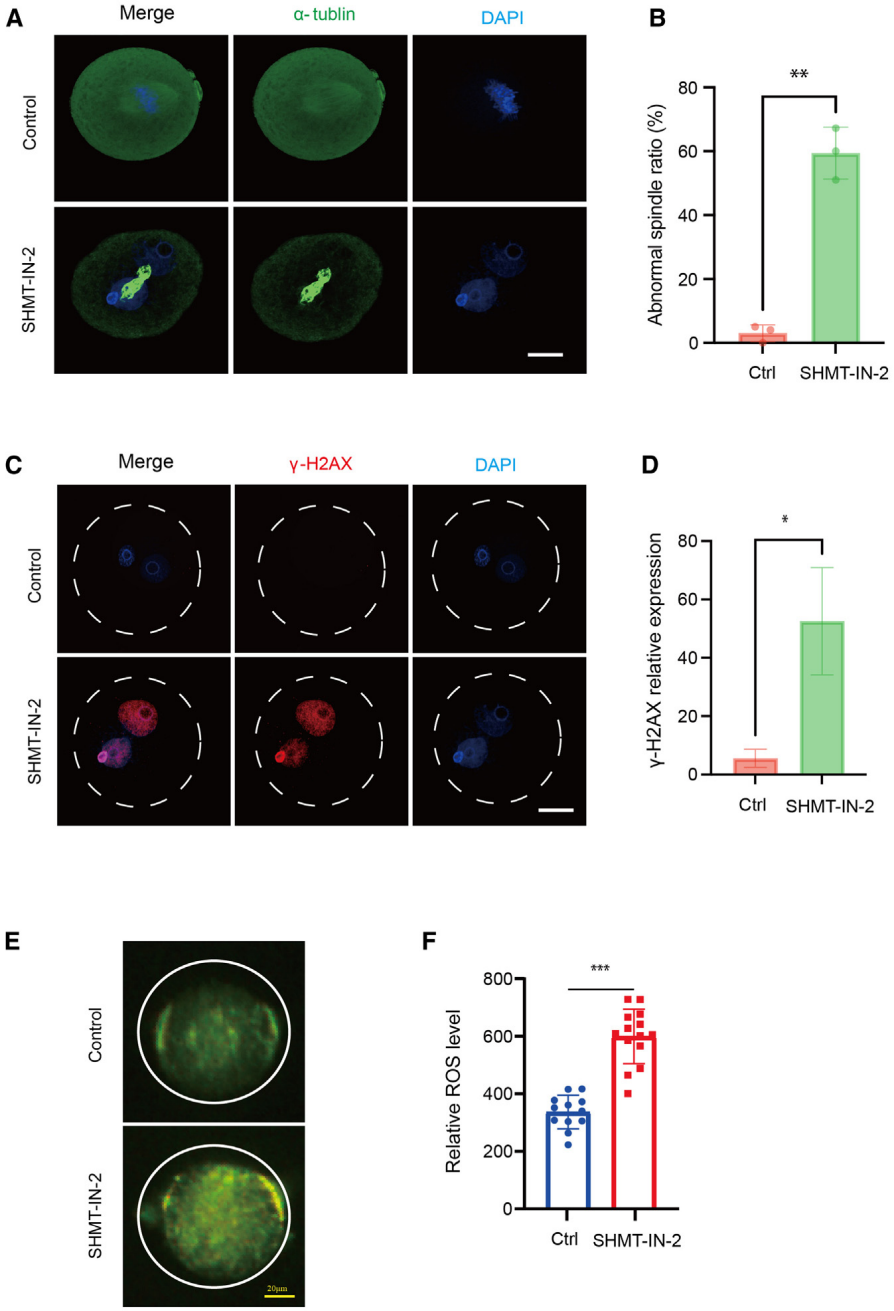
DNA replication stress induced by SHMT2 inhibition (A) Representative immunofluorescence images showing spindle morphology stained by α-tubulin and chromosome alignment in control and developmentally arrested embryos. Scale bar, 40 μm. (B) Quantification of abnormal spindle rate in the control and SHMT-IN-2-treated embryos. Data are from three independent experiments and are presented as the mean ± SEM. ∗∗, *p* < 0.01 (Student’s t test). (C) Representative immunofluorescence images showing DNA replication stress by γ-H2AX staining in control and developmentally arrested embryos. Scale bar, 40 μm. (D) Quantification of relative γ-H2AX expression in the control and SHMT-IN-2-treated embryos. Data are from three independent experiments and are presented as the mean ± SEM. ∗*p* < 0.05 (Student’s t test). (E) Representative images showing the control and SHMT-IN-2-treated zygotes with green fluorescence pixel intensity of ROS. Scale bar, 20 μm. (F) Relative ROS level (counts of photons) detection of single control (*n* = 12) and SHMT-IN-2-treated (*n* = 12) zygotes by optical nanoprobes. ∗∗∗*p* < 0.001 (Student’s t test).

After fertilization, male and female pronuclei normally undergo DNA replication during S phase. Followed by a short G2 phase, the first cleavage occurs after the completion of replication, where chromosomes are released from the pronuclei and captured by the mitotic spindle.^13^^,^^14^ Therefore, we hypothesized that the pronuclear fusion failure was due to incomplete DNA replication. Indeed, immunofluorescence staining showed that chromosomes did not properly align during metaphase II of the first mitotic cleavage, compared to the control, which formed metaphase plate (Figures 4A and 4B). A typical phenotype was that spindles were disorganized and stuck in the middle of the two pronuclei, reflecting spindle assembly defects and the failure of chromosome segregation (Figure 4A). In early embryonic cell divisions, incomplete DNA replication and replication stress are major causes of chromosome segregation errors.^15^ To confirm this possibility, we visualized sites of double-strand DNA breaks and found high levels of γ-H2AX signal in arrested SHMT2-inhibited embryos, indicating gross DNA damage and replication stress (Figures 4C and 4D).

Reactive oxygen species (ROS) maintain redox balance by their constant generation and elimination in the mitochondria. ROS are accumulated when they are generated excessively and thus induce oxidative stress, which has been proven to be one of the major factors harming developmental competence.^16^ Here, we tested ROS levels with a real-time single-cell multimodal analyzer equipped with a fiber probe. By using this single-living-cell detection system, we found that the ROS levels of SHMT2-inhibited embryos were significantly higher than those of control embryos at the zygote stage (Figures 4E and 4F), demonstrating that oxidative stress was induced by SHMT2 inhibition.

**Figure 5:**
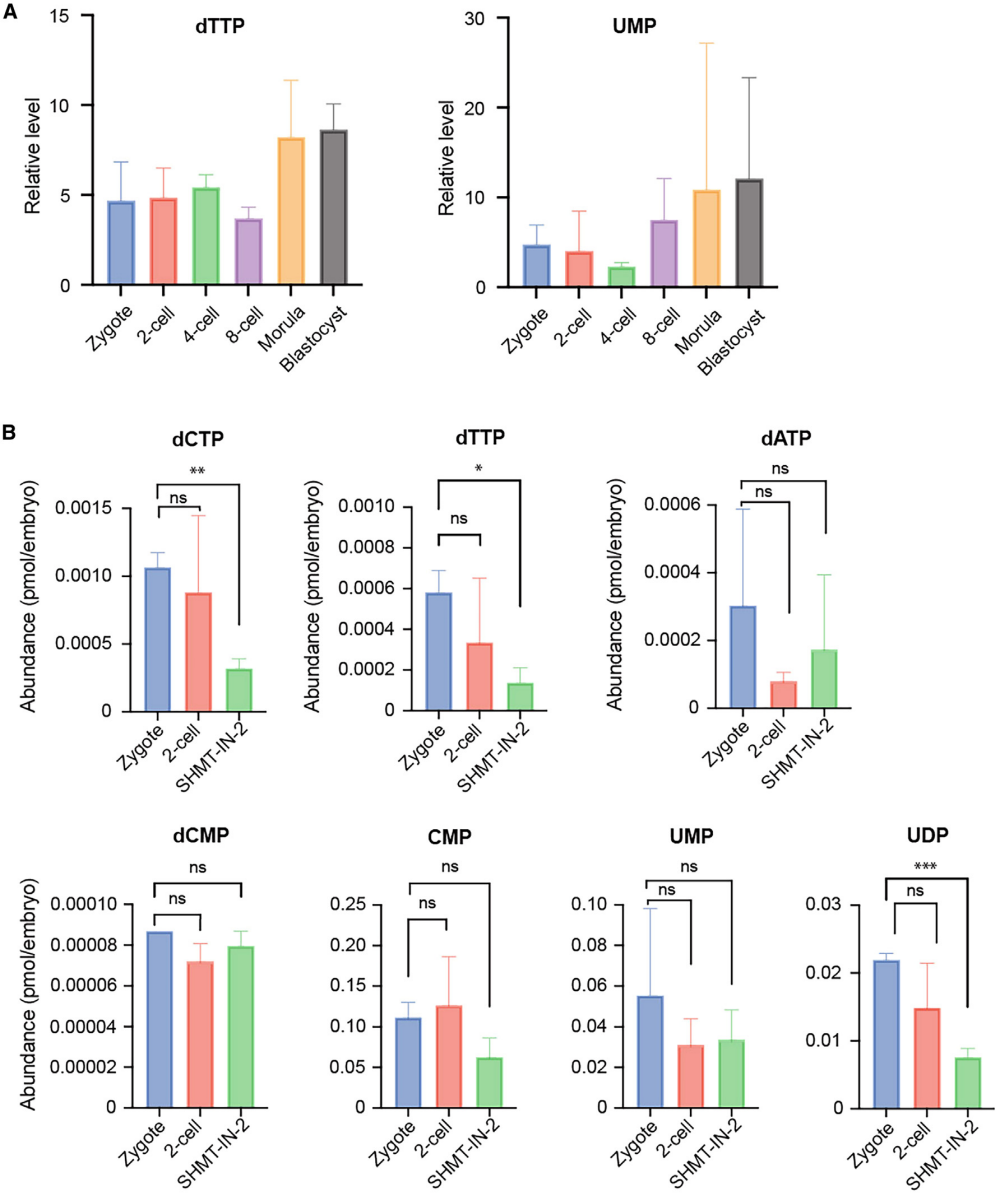
Levels of nucleotide metabolites measured by LC-MS/MS-based metabolomics (A) Analysis of previously published data^17^ from the untargeted metabolomics during mouse embryonic development. Each stage has six biological replicates. (B) The absolute contents of dCTP, dTTP, dATP, dCMP, CMP, UMP, and UDP per embryo were measured by liquid chromatography-tandem mass spectrometry (LC-MS/MS) in extracts from staged embryos (zygote, 19.5 h post hCG; 2-cell stage embryos, 28.5–30 h post hCG; SHMT-IN-2-treated embryos, 34.5–38 h post hCG). Data are from three independent experiments and are presented as the mean ± SEM. ∗*p* < 0.05, ∗∗*p* < 0.01, ∗∗∗*p* < 0.001 (Student’s t test).

To check whether the incomplete DNA replication and replication stress were due to the depletion of SHMT2 related compound of DNA, we intended to examine the dNTP levels in mouse early embryos. The nucleotide level at the zygote stage may reflect the maternally deposited precursors, whereas, at later stages, it may reflect the combination of maternal loaded and *de novo*-produced materials. Since nucleotide molecules are generally unstable compounds with high-energy phosphate bonds, their absolute levels have not yet been detected in mammalian embryos. Previously published data from untargeted metabolomics show the relative levels of nucleotides in mouse embryos from zygote to blastocyst stages.^17^ The relative dTTP levels remained steady from the zygote to the 8-cell stage, while the UMP levels showed a gentle decline from the zygote to the 4-cell stage (Figure 5A), indicating a balance between consumption and *de novo* biosynthesis.

To examine the absolute levels, we developed specific targeted liquid chromatography-tandem mass spectrometry (LC-MS/MS)-based metabolomics to analytically measure 7 nucleotides. Zygotes, 2-cell stage embryos, and SHMT2 inhibitor-treated arrested embryos were subjected to LC-MS/MS, and the results showed the consumption of dCTP, dTTP, and dATP from zygotes to 2-cell stage embryos during the first mitotic division, although not significant, indicating prompt *de novo* biosynthesis (Figures 5B, S2A, and S2B). Besides building blocks of DNA, dCMP, UDP, and UMP also showed insignificant consumption during the first mitotic division. Importantly, SHMT2 inhibition led to a significant depletion of dTTP, which aligned with its established role in the dTTP biosynthesis pathway. Moreover, SHMT2 inhibition also affected the levels of UDP and dCTP. The observed decrease in dCTP may be a consequence of misincorporation, potentially compensating for the reduced availability of dTTP,^18^ but this needs to be tested further. Interestingly, the abundance of CMP, UMP, and UDP was about 100 times higher than that of downstream dNTP, suggesting the uptake of substrates from the surrounding culture medium.

Taken together, these results indicate that SHMT2 inhibition interferes with dTMP biosynthesis and thus leads to dTTP depletion, and the insufficient supply of dTTP results in incomplete DNA replication during the first mitotic cleavage. Subsequent replication stress causes failure of pronuclear fusion and developmental arrest prior to the 2-cell stage.

### Disruption of proteomic characteristics for SHMT2-inhibited embryos

**Figure 6:**
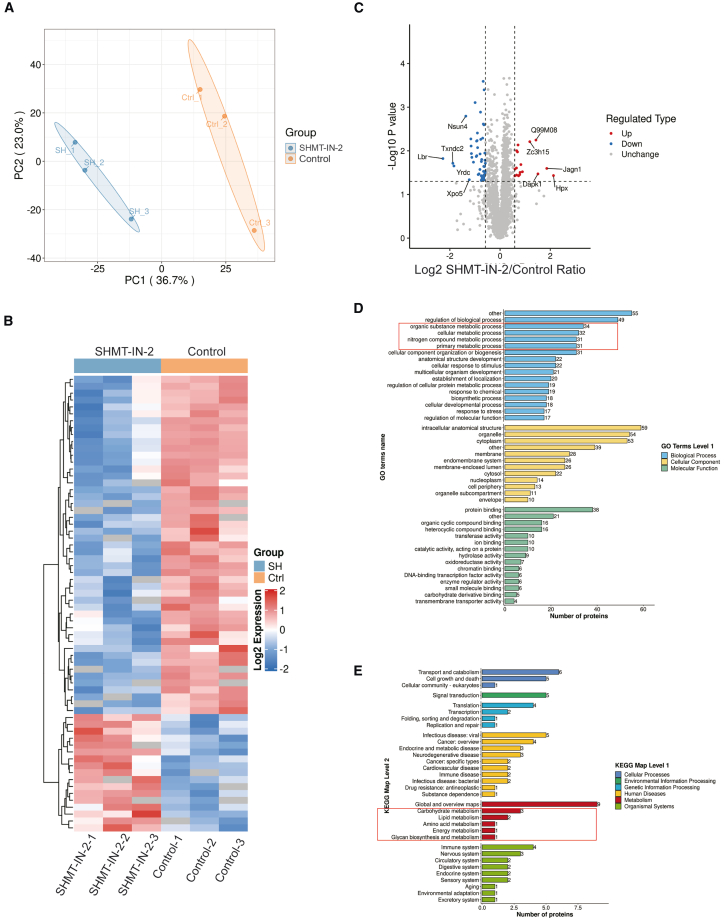
Proteomics characteristics for SHMT2-inhibited embryos (A) Principal-component analysis of samples from control (*n* = 3) and SHMT-IN-2-treated (*n* = 3) embryos. (B) Heatmap displaying differentially expressed proteins (DEPs) between control and SHMT-IN-2-treated embryos. Proteins that were expressed higher in SHMT-IN-2-treated embryos are shown in red, and proteins that were expressed lower are shown in blue. (C) Volcano map for expression levels of DEPs. Proteins that were upregulated in SHMT-IN-treated embryos are shown in red, and proteins that were downregulated are shown in blue. Unchanged proteins are shown in gray. (D) GO enrichment for DEPs in control and SHMT-IN-2-treated mouse embryos. Proteins enriched in pathways associated with metabolic processes are shown in a red box. (E) KEGG pathway for DEPs of control and SHMT-IN-2-treated embryos. Proteins enriched in pathways associated with metabolism are shown in a red box.

To further characterize global changes at the proteomic level after SHMT2 inhibition, we applied a high-sensitivity and speed of trapped ion mobility quadrupole time-of-flight mass spectrometer (timsTOF Pro) with low sample input to SHMT2 inhibitor-treated zygote embryos. Principal-component analysis showed that three biological replicates were statistically consistent within control and SHMT2-inhibited embryos, and the two groups could be clearly distinguished from each other (Figure 6A). In total, 3,671 proteins were identified and quantified, and 66 differentially expressed proteins (DEPs) were screened out by a log_2_ (fold change) greater than 1.3, among which 17 were upregulated and 49 downregulated, including YRDC, LBR, and XPO5 (Figures 6B and 6C). SHMT2 did not change at the protein level, as confirmed by western blot (Figure S2C).

The Gene Ontology (GO) analysis classified the DEPs into three categories: biological process, cellular component, and molecular function (Figure 6D). Notably, in terms of biological process, organic substance metabolic process, cellular metabolic process, nitrogen compound metabolic process, and primary metabolic process were shown to enrich 34, 32, 31, and 31 DEPs, respectively. The Kyoto Encyclopedia of Genes and Genomes (KEGG) classification system annotated DEPs in related biochemical pathways. We found a DEP enrichment in multiple metabolic pathways, including carbohydrate, lipid, amino acid, energy, and glycan biosynthesis (Figure 6E). These results indicate that metabolic processes were substantially altered due to SHMT2 inhibition.

## Discussion

Beyond basic functions as substrates of DNA building blocks, the availability of dNTP metabolites has been proven previously to be important in proliferation control of tumorigenesis.^19^^,^^20^ Various types of cancer, including melanoma, lung cancer, cervical cancer, and breast cancer, have been found to be associated with dNTP metabolism-related enzymes.^21^^,^^22^^,^^23^ Specifically, SHMT2 is required for glioma cell survival and promotes changes in metabolism that allow tumor cells to adapt to the environment; thus, preventing endogenous glycine production via SHMT2 inhibition is desired as an efficient therapy for tumorigenesis.^24^^,^^25^^,^^26^ On the other hand, dNTP levels correspond closely to other regulatory processes, such as DNA damage, genome instability, replication fidelity, and dNTP imbalance-caused misincorporation.^27^^,^^28^^,^^29^^,^^30^

The development of mammalian cleavage-stage embryos needs to be well regulated spatiotemporally with the input from multiple organizers.^31^ Early mitotic division mechanisms are acutely sensitive, and activation of cell cycle checkpoints is involved in a conserved way among species.^32^ Here, we found that inhibition of the SHMT2 enzyme led to developmental arrest prior to the 2-cell stage of the embryo. This arrest was due to an insufficient dNTP supply causing replication stress and subsequent failure of pronuclear fusion. Once fertilized, maternal and paternal DNA undergo replication within the pronuclear compartments and are then captured by the mitotic spindle when completing the replication.^14^^,^^15^^,^^33^ Limiting the dNTP supply may induce persisting DNA replication and thereby trigger activation of the replication checkpoint.^34^^,^^35^ We observed significant DNA replication stress and spindle assembly defects, in which the pronuclear phase was stuck and the first mitotic spindle was not able to segregate parental chromosomes.

Proteomics analysis characterized 3,671 proteins and 66 DEPs between control and SHMT2-inhibited embryos at the zygote stage. The DEPs were enriched in multiple metabolic pathways, indicating that metabolic processes were substantially altered by SHMT2 inhibition. Notably, the metabolic enzyme YRDC has been predicted to play roles in nucleotide metabolism by enabling nucleotidyltransferase and putative threonylcarbamoyl transferase activities.^36^ The Lamin B receptor (LBR), which is essential for cholesterol synthesis, has a role in gametogenesis and preimplantation development in that half of the zygotic LBR knockout embryos die before implantation.^37^^,^^38^^,^^39^ XPO5, a karyopherin protein, has been shown to be required for mouse embryonic development.^40^

Nucleotide molecules are generally unstable compounds with high-energy phosphate bonds; thus, targeted LC-MS/MS-based metabolomics with low input have yet to be well established. The absolute nucleotide levels in mammalian early embryos have not yet been quantitatively profiled. Here, we developed an optimized LC-MS/MS method with high sensitivity and found that dTTP levels remained unchanged in control embryos from the zygote to the 2-cell stage due to continuous biosynthesis, although with variation, consistent with previous data.^17^ As expected, dTTP levels showed significant depletion after SHMT2 inhibition, suggesting the absence of *de novo* biosynthesis when consumed. Furthermore, the absolute concentrations of dNTPs were detected, and we found that the zygote contained about 0.001 pmol/embryo maternal dCTP, 0.0006 pmol/embryo dTTP, and 0.0003 pmol/embryo dATP. This range is about 1,000 times lower than that in *Drosophila* oocytes, consistent with the divergence of their volumes.^41^ Furthermore, we observed an insignificant decline in most of the nucleotides from the zygote to the 2-cell stage embryos, showing a balance between consumption and *de novo* synthesis of nucleotides during the first mitotic cleavage.

Due to low abundance, we were not able to detect levels of dTMP in early embryos. It is notable that dTTP supplementation did not rescue the developmental arrest by SHMT2 inhibition, but dTMP partially rescued it. The biosynthesis of dTTP is from dTDP and its upstream substrate dTMP, and dTMP is the direct product from catalytic reaction of CH_2_-THF and dUMP. Little is known about functions of dTMP in embryogenesis apart from being a dTTP substrate. Thymidylate synthase (TYMS), the enzyme that catalyzes the conversion of CH_2_-THF and dUMP to dTMP, has been proven to be essential for early embryonic development.^42^ The TYMS mutant inner cell mass was unable to grow out from hatched embryos, indicating that the rapid cell division and DNA synthesis occurring in embryonic cells cannot be supported without normal TYMS function. Alternatively, it is possible that other regulatory pathways or epigenetic modifications were also perturbated when SHMT2 was inhibited and thus may induce an additive effect. We did not rule out the possibility that some side effects were present when using SHMT-IN-2.

Folate metabolism, in which SHMT and THF are involved, is crucial for embryogenesis, implantation, and early pregnancy and, thus, for the reduction of birth defect risks. The key component of folate metabolism is folic acid, which is required for DNA replication and acts as a substrate for a range of metabolic reactions, including nucleotide and amino acid biosynthesis. Adult women who are preparing for pregnancy are advised to supplement folic acid. Some folate metabolic enzymes, such as PHGDH, DHFR, and MTHFR, have been proven to be important for early development.^3^^,^^4^ Previous studies have shown that mice with SHMT1 overexpression and a folate-deficient diet exhibit a 10-fold increase in tissue nuclear uracil content compared with a 2-fold increase observed in wild-type mice on the same diet.^43^ Therefore, revealing the functions of SHMT during mouse preimplantation embryonic development is important for the understanding folate-related developmental arrest during the ART procedure and broadening the clinical options of the targeted therapies.

### Conclusion and future directions

**Figure 7:**
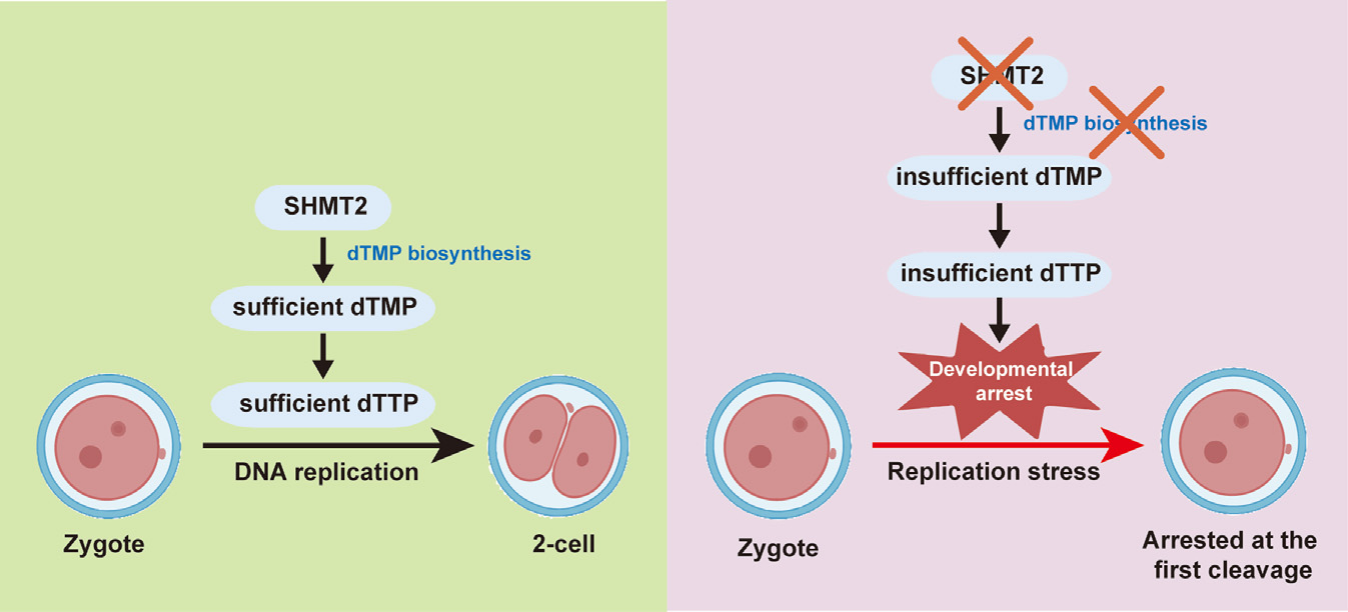
Schematic summary of the role of SHMT2 in early embryonic development When SHMT2 is present, sufficient dTMP and dTTP support DNA replication and further embryonic development. When maternally suppled dTMP and dTTP are exhausted, SHMT2 inhibition hinders their *de novo* biosynthesis, leading to an insufficient supply of dTTP and incomplete DNA replication during the first mitotic cleavage. Subsequent replication stress causes failure of pronuclear fusion and developmental arrest at the pronuclear stage.

In summary, inhibition of the metabolic enzyme SHMT2 results in developmental arrest of mammalian preimplantation embryos through interfering with cell cycle regulation. We demonstrate that maternally suppled dTMP and dTTP are exhausted during embryonic development and that SHMT2 inhibition hinders their *de novo* biosynthesis, leading to an insufficient supply of dTTP and incomplete DNA replication during the first mitotic cleavage. Subsequent replication stress causes failure of pronuclear fusion and developmental arrest prior to the 2-cell stage (Figure 7). Future work needs to be conducted to validate the mechanism in other models, and determine the effect of a folate-deficient diet and SHMT2 function in the crosstalk between the microenvironment and embryonic development. Animal testing as well as human trials need to be carried out to discover targeted therapies for metabolism-related developmental arrest during the ART procedure.

## Materials and methods

### Mouse embryo collection

All experiments conducted in this study were approved by the Ethics Committee of the Center for Reproductive Medicine of Shandong University. Male (13 weeks old) and female ICR mice (6–8 weeks old) were purchased from Vital River (Beijing, China) and bred under specific pathogen-free conditions and a 12 h light/dark cycle. Oocytes were collected from super-ovulated female mice treated with 6.5 IU of pregnant mare serum gonadotropin (PMSG) followed 45–47 h with 6.5 IU of human chorionic gonadotropin (hCG). Spermatozoa from the caudal epididymis and vas deferens of 13-week-old male mice were collected and capacitated in a 1.5 mL EP tube containing 1 mL G-IVF PLUS medium (10136, Vitrolife) for 45 min at 37°C and 5% CO_2_. Oocytes were collected 13.5 h post hCG treatment and transferred to 750 μL G-IVF PLUS medium, and capacitated spermatozoa were added with a final concentration of 0.25 × 106 cells/mL. After incubation at 37°C in the presence of 5% CO_2_ for 6 h, fertilized oocytes characterized by two pronuclei were cultured further in 500 μL G1 medium (10128, Vitrolife) at 37°C and 5% CO_2_. Embryonic development was observed on the fourth day of fertilization. The IVF procedure was identical in our routine practice and performed by experienced embryologists to reduce potential variability.

Embryos were staged at the following times post hCG injection and collected according to the developmental morphology: 24 h for the zygote stage, 34 h for the early 2-cell stage, 41 h for the middle 2-cell stage, 48 h for the late 2-cell stage, 58 h for the 4-cell stage, 69 h for the 8-cell stage, and 94 h for the blastocyst stage. Additionally, zygotes were collected at the following times: at 12 h for the PN0 stage, 15 h for PN1, 18 h for PN2, 21 h for PN3, 24 h for PN4, and 27 h for PN5.

### Treatment of embryos with inhibitors and chemicals

SHMT-IN-2 (HY-129226, MCE) was dissolved from powder to 10 mM concentrate with DMSO and diluted further to yield a final concentration of 95 μM in G1 culture medium. To assess the effects of SHMT-IN-2 on early embryonic development, after IVF, zygotes were cultured *in vitro* in G1 medium containing 95 μM SHMT-IN-2 for 3.5 h and then washed three times with G1 medium. Treated embryos were then cultured in G1 medium, and the relevant phenotypes were detected at the indicated time points under a microscope according to the morphology. To assess the rescue experiment, treated embryos were cultured *in vitro* in G1 medium containing 50 μM dTMP (R018, Thermo Scientific) or 50 μM dTTP (ab146540, Abcam), and the relevant phenotypes were detected under a microscope.

### Western blot

The embryos were lysed with 2× SDS loading buffer at 95°C for 5 min. The protein concentration was determined using a BCA kit (Thermo Fisher Scientific). The protein was separated by 10% SDS-PAGE and transferred to polyvinylidene fluoride membranes (Millipore). Blocking was performed for 1 h in 5% non-fat milk/PBS-T buffer, followed by incubation overnight with primary antibodies against SHMT2 (1:1,000 dilution, 33443, Cell Signaling Technology) and β-actin (HY-P80438, MCE) at 4°C. The next day, membranes were incubated with horseradish peroxidase-labeled goat anti-rabbit or anti-mouse H&L secondary antibodies (1:2,000 dilution, Zsbio) for 2 h at room temperature. The detection and analysis of the immunoreactive bands were performed using a ChemiDoc MP imaging system (Bio-Rad, USA).

### Immunofluorescence staining

After removing the zona pellucida with acidic operating fluid, mouse embryos were fixed in 4% polyformaldehyde in PBS (KGB5001, KeyGEN BioTECH) for 30 min, followed by permeabilization in 1% Triton X-100 (T8787, Sigma-Aldrich) for 30 min at room temperature. Embryos were then blocked in blocking solution (PBS containing 1% BSA, 0.1% Tween 20 [P9416, Sigma-Aldrich] and 0.01% Triton X-100) for 1 h at room temperature after three washes in washing solution (0.1% Tween 20 and 0.01% Triton X-100 in PBS). Embryos were incubated with antibodies for 1 h at room temperature, washed 3 times in washing solution, and then transferred to the secondary antibodies for 30 min. After being rinsed 3 times, embryos were transferred to the mounting medium (ab104135, Abcam). To assess the morphology of the spindle, embryos were incubated with fluorescein isothiocyanate-α-tubulin (1:500 dilution, F2168, Sigma). For evaluation of DNA damage, embryos were cultured with a histone H2AX phospho-Ser139 antibody (1:500 dilution, 39118, Proteintech) and donkey anti-rabbit immunoglobulin G (IgG) H&L (Alexa Fluor 594) (1:500 dilution, ab150064, Abcam). To capture the levels of SHMT2 between GV to blastocyst, oocytes and embryos were cultured with an SHMT2 antibody (1:500, TA808820, OriGene) and donkey anti-mouse IgG H&L (Alexa Fluor 488) (1:500 dilution, ab150109, Abcam). To capture the levels of SOX2 between GV to blastocyst, oocytes and embryos were cultured with a Sox2 antibody (1:200, 39843, Active Motif) and donkey anti-rabbit IgG H&L (Alexa Fluor 488) (1:500 dilution, ab150065, Abcam). To assess pronuclear fusion disorder, embryos were cultured with a Lamin A/C antibody (1:200 dilution, A0249, ABclonal), and chromosomes were counterstained with DAPI (1:500 dilution, D3571, Life Technologies). Images were captured under a laser-scanning confocal microscope (Dragonfly, Andor Technology, UK). Fluorescence images were processed using ImageJ (v.1.54). Nuclei were marked manually and identified as primary objects using the DAPI stain. The fluorescence intensities were plotted by normalization to DAPI or SOX2 using the plugin ImageJ function. The pronuclear fusion rate was quantified by determining the number of embryos that achieved 2-cell stage and the number of arrested embryos in which the two pronuclei stayed separately, and then it was divided by the number of total embryos in the field of view. The nuclear envelope formation rate was quantified by determining the LaminA/C-stained structure surrounding the nuclei that represented the nuclear membrane, and the number of embryos that showed a clear nuclear membrane was divided by the number of total embryos in the field of view.

### Analysis of ROS levels

To access the level of ROS in zygotes, the green fluorescent signal of ROS was detected using a ROS detection kit (DCFH-DA, D6883-50mg, Sigma). First, the zygotes were incubated at 37°C for 30 min in G1 droplets containing 20 μM DCFH-DA and then washed 3 times with G1 medium and transferred to Petri dishes containing G1 droplets for microscopy observation. The fluorescence intensity of a single zygote was measured using a real-time single-cell multimodal analyzer equipped with a fiber probe with ∼10 μm tips (Rayme, China). Within 30 s of inserting the photoprobe tip into the G1 droplet and aligning the zygote, photon counts were continuously detected and calculated.

### Metabolite extraction and targeted metabolomics analysis

Mouse embryos were collected in cold 80% methanol in 1.5 mL centrifuge tubes. 100 embryos were lysed in each tube and centrifuged at 15,000 × *g* for 15 min at 4°C, and the supernatants were transferred to a new pre-chilled tube. The supernatants were dried in a vacuum concentrator. Dried metabolites were resuspended in 100 μL water and centrifuged at 15 000 × *g* for 15 min at 4°C. LC-MS/MS was used to analyze the metabolites. The chromatography of 10 μL metabolite liquid was performed on an analytical HPLC system (Exion LC AD, SCIEX) with a Synergi 4 μm Fusion RP column (150 × 3 mm, Phenomenex), with mobile phase A consisting of 5 mM ammonium formate in water and mobile phase B consisting of 5 mM ammonium formate in acetonitrile. With a flow rate 0.25 mL/min, the following gradient was set: 0–2 min 1%–2% mobile phase B, 2–5 min 2–10% B, 5–8 min 10–90% B, 8–9 min 90% B, and 9–9 min 10 s 90%–1% B. MS was performed with a triple-quadrupole mass spectrometer (Triple Quad 6500+, SCIEX). The retention time of each metabolite was determined by injecting analytical standards into the LC-MS system. For quantification, the MultiQuant software was used. Technical support was provided by SCIEX.

### Proteomics

Mouse embryos of the control group and SHMT-IN-2 treatment group were collected, 100 each, into 1.5 mL centrifuge tubes with three biological replicates. Samples were sonicated three times on ice using a high-intensity ultrasonic processor in lysis buffer (8 M urea and 1% protease inhibitor cocktail) and centrifuged at 12,000 × *g* for 10 min at 4°C. The supernatants were transferred to a new tube, and the protein concentration was determined with a BCA kit according to the manufacturer’s instructions. For digestion, the protein solution was reduced with 5 mM dithiothreitol for 30 min at 56°C and alkylated with 11 mM iodoacetamide for 15 min at room temperature in darkness. The protein sample was then diluted by adding 200 mM TEAB to a urea concentration of less than 2 M. Trypsin was added at a 1:50 trypsin-to-protein mass ratio for the first digestion overnight and a 1:100 trypsin-to-protein mass ratio for a second 4 h digestion. Finally, the peptides were desalted by a Strata X SPE column. The tryptic peptides were dissolved in solvent A, directly loaded onto a home-made reverse-phase analytical column (25-cm length, 100 μm). The mobile phase consisted of solvent A (0.1% formic acid and 2% acetonitrile/in water) and solvent B (0.1% formic acid in acetonitrile). Peptides were separated with following gradient: 0–70 min 6%–24% B, 70–82 min 24%–35% B, 82–86 min 35%–80% B, and 86–90 min 80% B, all at a constant flow rate of 450 nL/min on a NanoElute UHPLC system (Bruker Daltonics). The peptides were subjected to a capillary source followed by timsTOF Pro mass spectrometry. The electrospray voltage was 1.75 kV. Precursors and fragments were analyzed at the TOF detector with an MS/MS scan range from 100 to 1,700. The timsTOF Pro was operated in parallel accumulation serial fragmentation (PASEF) mode. Precursors with charge states 0–5 were selected for fragmentation, and 10 PASEF MS/MS scans were acquired per cycle. The dynamic exclusion was set to 30 s. The resulting MS/MS data were processed using the MaxQuant search engine (v.1.6.15.0). Tandem mass spectra were searched against Mus_musculus_10090_SP_20230103.fasta (17,132 entries) concatenated with the reverse decoy and contaminants database.

### Statistical analysis

Statistical analyses were conducted with GraphPad Prism software (v.9.3, GraphPad, CA, USA) with t tests and one-way ANOVA. For each experiment, at least three replicates were performed, and the results were expressed as mean ± SEM. *p* < 0.05 was considered significant (∗*p* < 0.05, ∗∗*p* < 0.01, ∗∗∗*p* < 0.001).

## Data availability

The data that support the findings of this study are available from the corresponding author upon reasonable request.

## Acknowledgments

This work was supported by the 10.13039/501100001809National Natural Science Foundation of China (32100650), the 10.13039/501100007129Shandong Provincial Natural Science Foundation (2022HWYQ-034 and ZR2021QC106), the Taishan Scholars Program of Shandong Province (tsqn202211373), Instrument Improvement Funds of the Shandong University Public Technology Platform (ts20230206), 10.13039/501100012226Fundamental Research Funds for the Central Universities (2022JC006), and the CAMS Innovation Fund for Medical Sciences (2021-I2M-5-001). The authors thank Junmiao Chen and Yanhui Gao for technical support.

## Author contributions

M.S., Y.H., and T.D. made substantial contributions to the acquisition, analysis, and interpretation of data. C.Z., J.S., J.W., and Y.Z. made substantial contributions to technical support. Z.-J.C., H.Z., and K.W. made substantial contributions to revising the manuscript. B.L. made substantial contributions to the conception and design of the work and drafting of the manuscript.

## Declaration of interests

The authors declare no competing interests.

## Supplemental information

Document S1. Figures S1 and S2

Document S2. Article plus supplemental information
