# Supplementary material for: SHMT2 is essential for mammalian preimplantation embryonic development through de novo biosynthesis of nucleotide metabolites
Source: Mol Ther Nucleic Acids. 2025 Mar 5;36(2):102499. doi: 10.1016/j.omtn.2025.102499 (PMC11960634; doi:10.1016/j.omtn.2025.102499)
Supplement: Document S1. Figures S1 and S2 [file mmc1.pdf]

OMTN, Volume *36*

Supplemental information

SHMT2 is essential for mammalian preimplantation embryonic development through *de novo* biosynthesis of nucleotide metabolites

Mingze Shi, Yingxue Huai, Tiantian Deng, Chuanxin Zhang, Jinzhu Song, Jiawei Wang, Yiwen Zhang, Zi-Jiang Chen, Han Zhao, Keliang Wu, and Boyang Liu

Supplemental figures

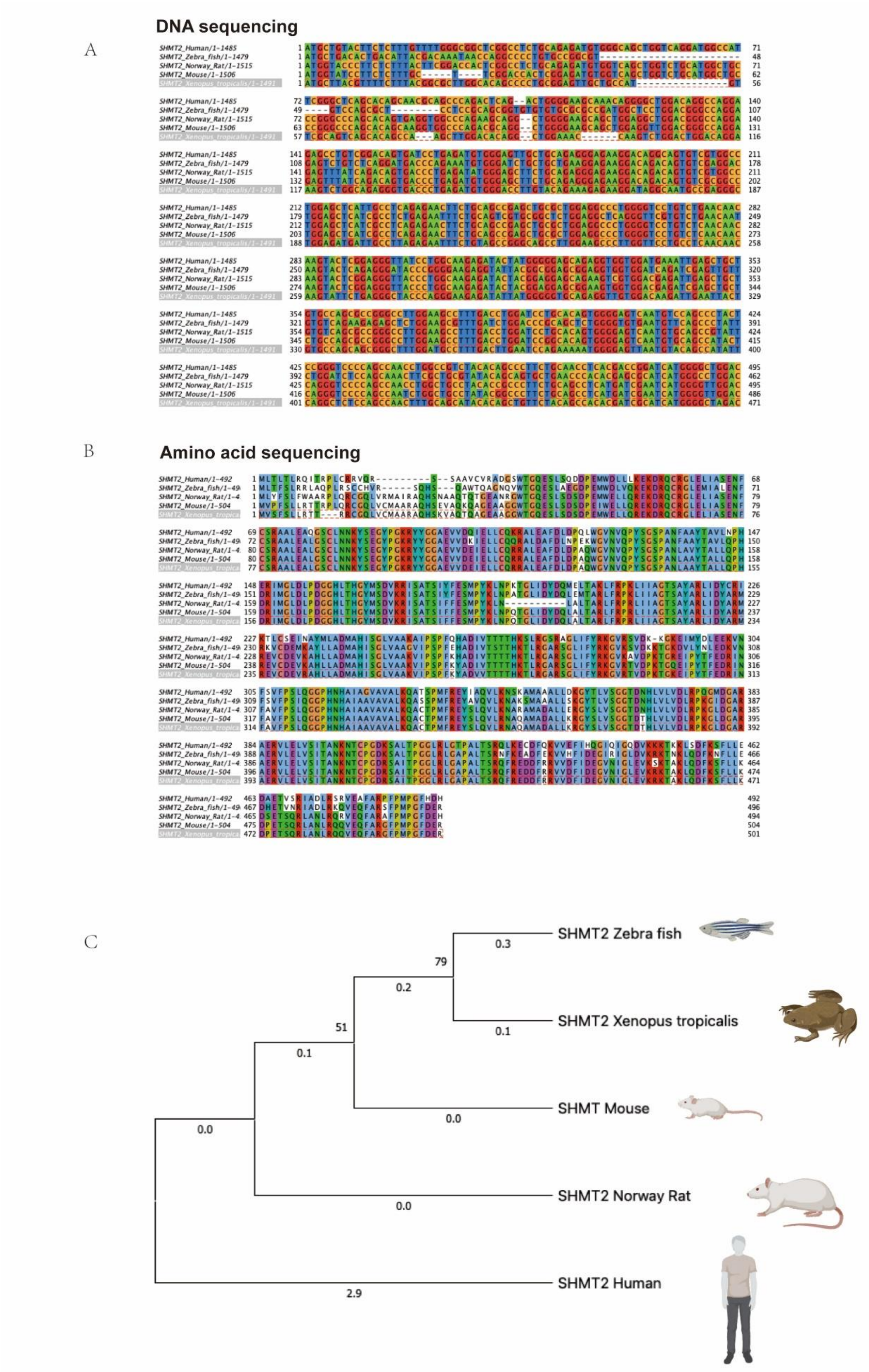

Figure S1. SHMT2 is conserved among species.

(A-B) Comparison and alignment of DNA and amino acid sequencing of SHMT2 among human, mouse, zebrafish, Norway rat and Xenopus tropicalis.

(C) SHMT2 evolutionary trees among human, mouse, zebrafish, Norway rat and Xenopus tropicalis. The longer the branch in the horizonal dimension, the larger the amount of change. Numbers indicate the length of the branch that represents an amount genetic change.

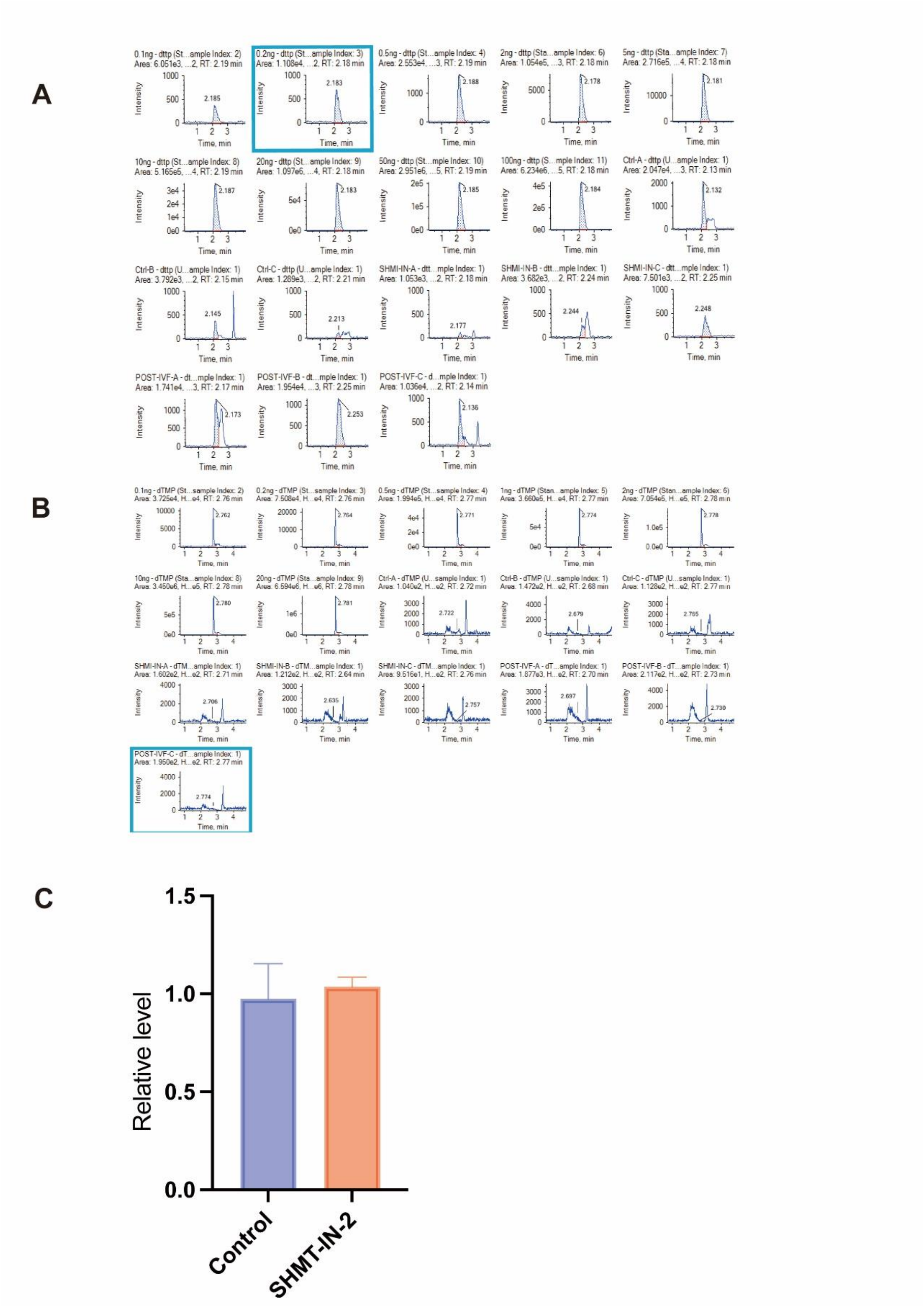

Figure S2. Chromatographic peak area of representative nucleotide metabolites.

(A-B) dTTP and dTMP LC-MS/MS chromatographic peaks.

(C) Proteomics showed no change in SHMT2 protein level after the treatment of SHMT-IN-2.
